# Supplementary material for: Biodegradation of Poly(ethylene terephthalate) by Bacillus safensis YX8
Source: Int J Mol Sci. 2023 Nov 17;24(22):16434. doi: 10.3390/ijms242216434 (PMC10671283; doi:10.3390/ijms242216434)
Supplement: Supplementary file 1 [file ijms-24-16434-s001.zip › ijms-2694879-supplementary.pdf]

Supplementary Materials for

Biodegradation of Poly (Ethylene Terephthalate) by *Bacillus safensis* YX8

Caiting Zeng [1], Fanghui Ding [1], Jie Zhou [2], Weiliang Dong [2], Zhongli Cui [1] and Xin Yan [1,3,*]

1 College of Life Sciences, Nanjing Agricultural University, Nanjing 210095, China; 2020116069@stu.njau.edu.cn (C.Z.); 2022116053@stu.njau.edu.cn (F.D.); czl@njau.edu.cn (Z.C.)

2 College of Biotechnology and Pharmaceutical Engineering, Nanjing Tech University, Nanjing 211816, China; jayzhou@njtech.edu.cn (J.Z.); dwl@njtech.edu.cn (W.D.)

3 Jiangsu Provincial Key Laboratory for Organic Solid Waste Utilization, Nanjing Agricultural University, Nanjing 210095, China

* Correspondence: yanxin@njau.edu.cn

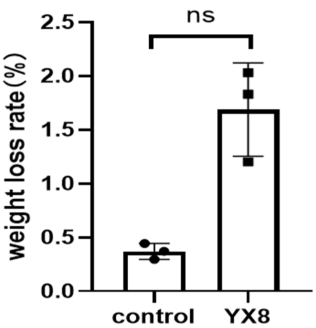

Figure S1. The weight loss rate of PET film after incubated with or without strain YX8. One (2 cm×2 cm) PET film was incubated in 50mL LB broth with or without strain YX8 at 45℃ and 200 rpm for 2 months.

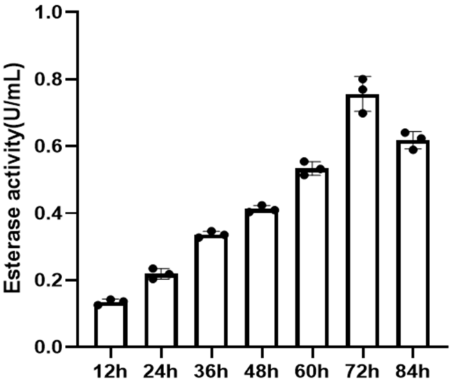

Figure S2. The extracellular esterase activity of strain YX8 grown in LB broth. The esterase assay system included 10 μL of 10 mM *p*-nitrophenol acetate, 10 μL of crude enzyme, 980 μL of 50 mM Tris-HCl (pH 8.0 and 300 mM NaCl). The reaction was carried out at 25℃ for 3 min, and then OD_{405} was detected by spectrophotometer.

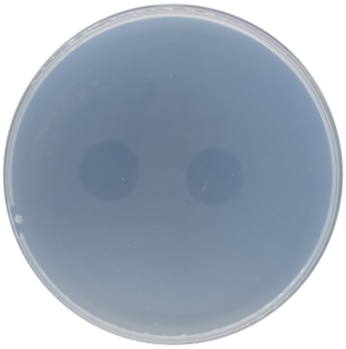

Figure S3. Detection the PCL-hydrolyzing activity of concentrated crude enzymes. Strain YX8 was grown in LB broth for 72 h. The extracellular enzymes were concentrated and dropped on LB plate containing 0.44% PCL.

Table S1. Reported PET-degrading strains and enzymes.

Enzyme	Source	Substrate	PET degradation ability and temperature	Reference
PET2	Uncultured bacterium (marine metagenome)	nanoparticle ager	Zone of clearance, 50℃	[1]
PET5	*Oleispira antarctica* RB-8	nanoparticle ager	Zone of clearance , 50℃	[1]
PET12	*Polyangium brachysporum*	nanoparticle ager	Zone of clearance , 50℃	[1]
PET6	*Vibrio gazogenes*	nanoparticle ager	Zone of clearance , 50℃	[1]
PE-H	*Pseudomonas aestusnigri* VGXO14^T	amorphous PET film	MHET released in 48 h, 30℃	[2]
CALB	*Candida antarctica*	Low-crystallinity and biaxially oriented PET films	TPA, BHET, MHET were released	[3]
LCC	Metagenome from leaf branch compost	amorphous PET film	50% weight loss, 50-70℃	[4]
LCC^{ICCG}	Engineered LCC	amorphous PET film	90% weight loss in 9.3 h, 72℃	[5]
Hic	*Humicola insolens*	lcPET (crystallinity 7%)	97 ± 3% weight loss in 6 day, 70℃	[6]
FsC	*Fusarium solani pisi*	lcPET (crystallinity 7%)	5% weight loss in 4 days, 30-60℃	[6]
Cut190	*Saccharomonospora viridis*AHK190	Amorphous PET film and package-grade PET	TPA and MHET released in 3 days,60-65℃	[7]
TfH	*Thermobififida fusca* DSM43793	bottle-grade PET (crystallinity 10 %)	50% weight loss, 55℃	[8]
Thc_Cut1	*Thermobifida cellulosilytica* DSM44535	PET film (crystallinity 37%)	MHET, TPA, and HEB released in 5 days, 50℃	[9]
Thc_Cut2	*Thermobifida cellulosilytica* DSM44535	PET film (crystallinity 37%)	MHET, TPA, and HEB released in 5 days, 50℃	[9]
Thf42_Cut1	*Thermobifida fusca* DSM44342	PET film (crystallinity 37%)	MHET, TPA, and HEB released in 5 days, 50℃	[9]
TfCut2	*Thermobifida fusca* KW3	PET film	12.6% weight loss in 48 h, 55-65℃	[10]
Tcur0390	*Thermomonospora curvata* DSM43183	PET nanoparticle	20 μg/mL substrate degrade rate 5.9×10^{-3} min^{-1},50℃	[11]
TfCa	*Thermobifida fusc* KW3	cyclic PET trimers	EMT, MHET, and BHET were released, 50-60℃	[12]
BsEstB	*Bacillus subtilis*	3PET	TPA, MHET and BA were released, 40-45℃	[13]
Thh_Est	*Thermobifida halotolerans* DSM44931	3PET	TPA, BA HEB, and MHET were released, 50℃	[14]

IsPETase	*Ideonella sakaiensis* 201-F6	PET film (crystallinity 1.9%)	TPA, MHET, and EG released in 1 day, 20-45℃	[15]
Unknown	*Bacillus safensis* YX8	PET nanoparticles	TPA, MHET, and BHET released in 48h, 45℃	This study

Reference

1. Danso D, Schmeisser C, Chow J, et al. New Insights into the Function and Global Distribution of Polyethylene Terephthalate (PET)-Degrading Bacteria and Enzymes in Marine and Terrestrial Metagenomes. Appl. Environ. Microbiol. 2018, 84(8): e02773-17.
2. Bollinger A, Thies S, Knieps-Grünhagen E, et al. A Novel Polyester Hydrolase From the Marine Bacterium Pseudomonas aestusnigri - Structural and Functional Insights. Front. Microbiol. 2020, 11: 114.
3. Carniel A, Valoni É, Nicomedes J, et al. Lipase from Candida antarctica (CALB) and cutinase from Humicola insolens act synergistically for PET hydrolysis to terephthalic acid. Process Biochem. 2017, 59: 84-90.
4. Sulaiman S, Yamato S, Kanaya E, et al. Isolation of a novel cutinase homolog with polyethylene terephthalate-degrading activity from leaf-branch compost by using a metagenomic approach. Appl. Environ. Microbiol. 2012, 78(5): 1556-62.
5. Tournier V, Topham C M, Gilles A, et al. An engineered PET depolymerase to break down and recycle plastic bottles. Nature. 2020, 580(7802): 216-219.
6. Ronkvist Å M, Xie W, Lu W, et al. Cutinase-Catalyzed Hydrolysis of Poly(ethylene terephthalate). Macromolecules. 2009, 42(14): 5128-5138.
7. Kawai F, Oda M, Tamashiro T, et al. A novel Ca^{2+}-activated, thermostabilized polyesterase capable of hydrolyzing polyethylene terephthalate from Saccharomonospora viridis AHK190. Appl. Microbiol. Biotechnol. 2014, 98(24): 10053-64.
8. Müller R J, Schrader H C G, Profe J, et al. Enzymatic Degradation of Poly(ethylene terephthalate): Rapid Hydrolyse using a Hydrolase from T. fusca. Macromol Rapid Commun. 2005, 26: 1400-1405.
9. Herrero Acero E, Ribitsch D, Steinkellner G, et al. Enzymatic Surface Hydrolysis of PET: Effect of Structural Diversity on Kinetic Properties of Cutinases from Thermobifida. Macromolecules. 2011, 44(12): 4632-4640.
10. Then J, Wei R, Oeser T, et al. Ca2+ and Mg2+ binding site engineering increases the degradation of polyethylene terephthalate films by polyester hydrolases from Thermobifida fusca. BIOTECHNOL J. 2015, 10(4): 592-598.
11. Wei R, Oeser T, Then J, et al. Functional characterization and structural modeling of synthetic polyester-degrading hydrolases from Thermomonospora curvata. AMB Express. 2014, 4: 44.
12. Billig S, Oeser T, Birkemeyer C, et al. Hydrolysis of cyclic poly(ethylene terephthalate) trimers by a carboxylesterase from Thermobifida fusca KW3. Appl. Microbiol. Biotechnol. 2010, 87(5): 1753-64.

13. Ribitsch D, Heumann S, Trotscha E, et al. Hydrolysis of polyethyleneterephthalate by p-nitrobenzylesterase from Bacillus subtilis. Biotechnol. Prog. 2011, 27(4): 951-60.
14. Ribitsch D, Herrero Acero E, Greimel K, et al. A New Esterase from Thermobifida halotolerans Hydrolyses Polyethylene Terephthalate (PET) and Polylactic Acid (PLA). Polymers. 2012, 4(1): 617-629.
15. Yoshida S, Hiraga K, Takehana T, et al. A bacterium that degrades and assimilates poly(ethylene terephthalate). Science. 2016, 351(6278): 1196-9.

16s rRNA gene sequence

GGCGGCGTGCCTAATACATGCAAGTCGAGCGGACAGAAGGGAGCTTGCTCCCGGATGTTAGCGGCGGACGG
GTGAGTAACACGTGGGTAACCTGCCTGTAAGACTGGGATAACTCCGGGAAACCGGAGCTAATACCGGATAGT
TCCTTGAACCGCATGGTTCAAGGATGAAAGACGGTTTCGGCTGTCACTTACAGATGGACCCGCGGCGCATTA
GCTAGTTGGTGGGGTAATGGCTCACCAAGGCGACGATGCGTAGCCGACCTGAGAGGGTGATCGGCCACACTG
GGACTGAGACACGGCCCAGACTCCTACGGGAGGCAGCAGTAGGGAATCTTCCGCAATGGACGAAAGTCTGA
CGGAGCAACGCCGCGTGAGTGATGAAGGTTTTCGGATCGTAAAGCTCTGTTGTTAGGGAAGAACAAGTGCGA
GAGTAACTGCTCGCACCTTGACGGTACCTAACCAGAAAGCCACGGCTAACTACGTGCCAGCAGCCGCGGTA
ATACGTAGGTGGCAAGCGTTGTCCGGAATTATTGGGCGTAAAGGGCTCGCAGGCGGTTTCTTAAGTCTGATGT
GAAAGCCCCCGGCTCAACCGGGGAGGGTCATTGGAAACTGGGAAACTTGAGTGCAGAAGAGGAGAGTGGA
ATTCCACGTGTAGCGGTGAAATGCGTAGAGATGTGGAGGAACACCAGTGGCGAAGGCGACTCTCTGGTCTGT
AACTGACGCTGAGGAGCGAAAGCGTGGGGAGCGAACAGGATTAGATACCCTGGTAGTCCACGCCGTAAACG
ATGAGTGCTAAGTGTTAGGGGGTTTCCGCCCCTTAGTGCTGCAGCTAACGCATTAAGCACTCCGCCTGGGGAG
TACGGTCGCAAGACTGAAACTCAAAGGAATTGACGGGGGCCCGCACAAGCGGTGGAGCATGTGGTTTAATT
CGAAGCAACGCGAAGAACCTTACCAGGTCTTGACATCCTCTGACAACCCTAGAGATAGGGCTTTCCCTTCGG
GGACAGAGTGACAGGTGGTGCATGGTTGTCGTCAGCTCGTGTCGTGAGATGTTGGGTTAAGTCCCGCAACGA
GCGCAACCCTTGATCTTAGTTGCCAGCATTCAGTTGGGCACTCTAAGGTGACTGCCGGTGACAAACCGGAGG
AAGGTGGGGATGACGTCAAATCATCATGCCCCTTATGACCTGGGCTACACACGTGCTACAATGGACAGAACA
AAGGGCTGCAAGACCGCAAGGTTTAGCCAATCCCATAAATCTGTTCTCAGTTCGGATCGCAGTCTGCAACTCG
ACTGCGTGAAGCTGGAATCGCTAGTAATCGCGGATCAGCATGCCGCGGTGAATACGTTCCCGGGCCTTGTACA
CACCGCCCGTCACACCACGAGAGTTTGCAACACCCGAAGTCGGTGAGGTAACCTTTATGGAGCCAGCCGCC
GAAGGTGG

gyrA gene sequence

GGCGCCCTTCCAGTTCATGGATCTTCGTGATATCACCAAGGATACGATTGATTATCAAGATAACTACGATGGCA
GTGAAAGAGAACCAATCGTCATGCCTGCTCGTTTCCCGAACTTATTAGTCAACGGGGCAACAGGTATCGCTGT
AGGGATGGCAACCAACATTCCGCCTCACCAGCTAGGTGAAGTGATTGATGGGGTACTGGCGATAAGTCAAAA
CCCAGAAATGACAACACAGGAACTAATGGAGATCATTCCAGGACCAGACTTCCCGACTGCGGGACAAATCA
TCGGCCGAAGCGGAATCCGCAAAGCATATGAAACAGGCCGGGGCTCTATTACATTACGTGCAAATTCTATTAT
TGAAGAAACATCCAGCGGTAAACAGCGGATTGTCATCAATGAAATTCCATATCAGGTCAACAAAGCACGTTT
GATCGAAAAAATCGTGGATCTTGTAA
